# Randomized-controlled trial of sacubitril/valsartan and amlodipine combination in Japanese uncontrolled hypertensive patients (J-SAMIT)

**DOI:** 10.1038/s41440-026-02704-7

**Published:** 2026-06-30

**Authors:** Kazuomi Kario, Hiromi Rakugi, Chiyo Ito, Sarfaraz Sayyed, Toshihito Kitamura, Yukina Tanaka, Ankur Malhotra

**Affiliations:** 1 Division of Cardiovascular Medicine, Department of Medicine, Jichi Medical University School of Medicine, Tochigi, Japan; 2 Osaka Rosai Hospital, Sakai, Osaka Japan; 3 Novartis Pharma K.K., Tokyo, Japan; 4 Novartis Healthcare Private Limited, Mumbai, India

**Keywords:** ARNI, CCB, Combination therapy, Hypertension, Randomized-controlled trial

## Abstract

This multicenter, randomized, double-blind, parallel-group, active-controlled study aimed to demonstrate a superior BP-lowering effect of sacubitril/valsartan (SacVal) and amlodipine (AML) combination therapy vs SacVal monotherapy in Japanese patients with grade Ⅰ/Ⅱ essential hypertension inadequately controlled with SacVal monotherapy. After 4 weeks of single-blind active run-in with SacVal 200 mg monotherapy, patients (n=717) with mean sitting systolic blood pressure (msSBP) of 140 to <180 mmHg were randomized and received SacVal 200 mg monotherapy or SacVal 200 mg in combination with AML (2.5, 5, or 10 mg) for 8 weeks. All SacVal/AML 200 mg/2.5 mg, 200 mg/5 mg, 200 mg/10 mg combinations achieved statistically significant, dose-dependent reductions in msSBP vs SacVal 200 mg monotherapy (treatment difference [mmHg] [95% CI]: −5.63 [ −8.01, −3.25], −12.94 [ −15.22, −10.67], −16.39 [ −19.01, −13.77], respectively; all p < 0.001). Treatment effects of all combinations were also statistically significant in 24-h ambulatory SBP (treatment difference [mmHg] [95% CI] vs SacVal: −6.48 [ − 8.79, −4.18], −14.18 [ −16.63, −11.73], −17.70 [ −20.24, −15.26], respectively; all p < 0.001) and BP control ( < 140/90 mmHg) rates (57.2, 72.5, 79.9%, respectively, vs 35.0% in SacVal with odds ratio [95% CI]: 2.16 [1.40, 3.33], 5.28 [3.31, 8.43], 7.23 [4.41, 11.86], respectively; all p < 0.001). Subgroup and waterfall plot analyses in msSBP demonstrated consistent BP-lowering effects across patient demographic and baseline characteristics. The safety profile of combination therapy was similar to that of SacVal with no new safety findings. These results demonstrated that combination therapy provided superior BP reduction with favorable safety and tolerability in Japanese patients inadequately controlled by SacVal 200 mg monotherapy.

**Figure:**
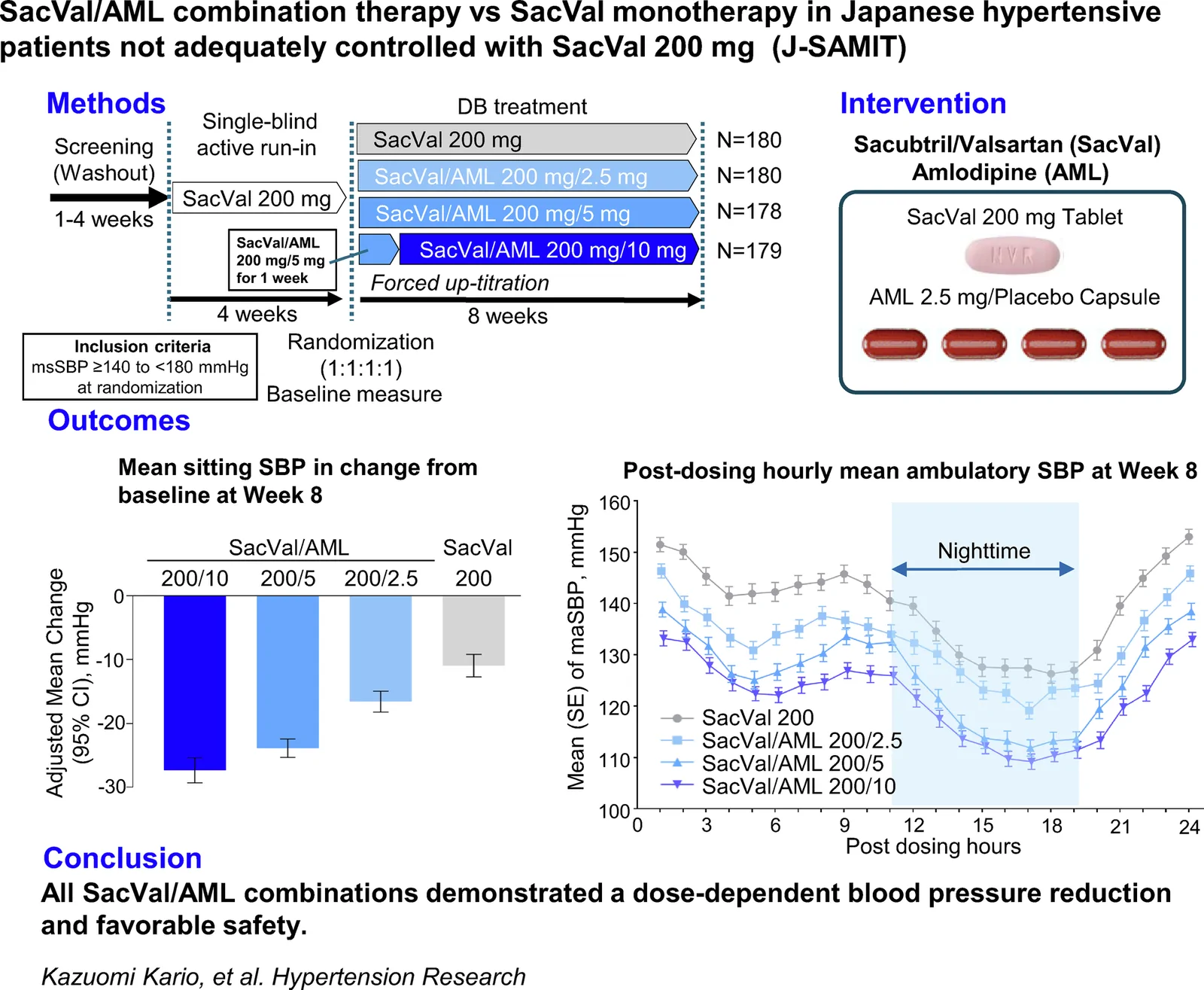
In this multicenter, randomized, double-blind, parallel group, active controlled trial, sacubitril/valsartan and amlodipine combination therapy showed dose-dependent reductions in office and 24-hour systolic blood pressure and a favourable safety profile in Japanese patients inadequately controlled on sacubitril/valsartan monotherapy, supporting its efficacy and safety as a treatment option

In this multicenter, randomized, double-blind, parallel group, active controlled trial, sacubitril/valsartan and amlodipine combination therapy showed dose-dependent reductions in office and 24-hour systolic blood pressure and a favourable safety profile in Japanese patients inadequately controlled on sacubitril/valsartan monotherapy, supporting its efficacy and safety as a treatment option

## Introduction

Hypertension, the leading cause of cardiovascular disease in Japan, affects an estimated 43 million Japanese people and causes approximately 170,000 cardiovascular deaths annually [1]. The goals of antihypertensive therapy are to prevent the occurrence, progression, and recurrence of cardiovascular diseases and reduce mortality [1].

Based on accumulated evidence, the current Japanese Society of Hypertension guideline (JSH2025) recommends a target blood pressure (BP) < 130/80 mmHg regardless of age or comorbidities [2]. However, BP control remains inadequate in Japan, despite the availability of various classes of antihypertensive drugs. Indeed, a large-scale longitudinal study in Japan found that less than 50% of patients receiving antihypertensive drugs had controlled BP ( < 140/90 mmHg) [1] and only approximately 20% achieved the current target BP < 130/80 mmHg [3]. Further, the BP control rate ( < 140/90 mmHg) has been reported to be lower in Japan than in other high-income countries [4, 5]. Data from a recent study suggests that the proportion of patients with uncontrolled BP ( ≥ 140/90 mmHg) with monotherapy alone was approximately 50% [6]. In addition, despite nocturnal and morning hypertension being positioned as important risk factors for cardiovascular disease [7–9], nocturnal and morning BP are difficult to control with 45-55% of patients who received even 2 or 3 antihypertensive drugs remain uncontrolled [6], resulting in poor cardiovascular outcomes [10]. Therefore, to achieve the target BP ( < 130/80 mmHg) and nocturnal/morning BP control, more effective combination therapy is needed in many patients.

Sacubitril/valsartan (SacVal), an angiotensin receptor neprilysin inhibitor (ARNI), provides renin-angiotensin-aldosterone system (RAAS) suppression and neprilysin inhibition simultaneously [11]. RAAS suppression exerts antihypertensive effects by inhibiting angiotensin II-induced potent vasoconstriction, fluid retention, and sympathetic nerve activation through angiotensin II type 1 (AT_1_) receptor antagonism. Neprilysin inhibition inhibits the degradation of bioactive natriuretic peptides (NPs), resulting in increased endogenous NPs and exerts pleiotropic effects including vasodilatory and natriuretic effects [12]. Amlodipine (AML) is widely used as a representative calcium channel blocker (CCB) that acts on vascular smooth muscle cells and exert vasodilation-induced antihypertensive effect [1]. Combination therapy with different classes of antihypertensive drugs is considered to be a reasonable and desirable approach to achieve target BP levels safely. The combination of an angiotensin II receptor blocker (ARB) with a CCB is the most recommended combination therapy according to the JSH2025 guideline [1]. AML increases the dependence of RAAS for BP regulation [13, 14] and as a result the antihypertensive effect of AT_1_ receptor antagonism of an ARB may be enhanced by combination with AML. Combination therapy of ARB and AML is expected to reduce peripheral edema [15, 16], a characteristic adverse reaction of AML. As SacVal has an AT_1_ receptor antagonistic effect, the combination of SacVal and AML is reasonably expected to enhance antihypertensive effect and may reduce adverse reactions typically observed with AML.

It has been previously reported that SacVal added to AML demonstrated a superior BP-lowering effect in Asian patients not adequately controlled with AML monotherapy [17]. However, no studies have evaluated the effect of adding AML in patients who are not adequately controlled with SacVal monotherapy.

This study in Japanese patients not adequately controlled by SacVal monotherapy comprised an 8-week randomized double-blind (DB) part to evaluate the efficacy and safety of SacVal/AML combination therapy compared to SacVal monotherapy and a 52-week open-label extension (OLE) part to evaluate the long-term safety, tolerability, and efficacy of SacVal/AML combination therapy (OLE part). This paper focuses on the DB part.

## Methods

### Study design and patients

This multicenter, randomized, double-blind, parallel-group, active-controlled study (ClinicalTrials.gov: NCT06236061) was composed of a DB part that is the focus of this report, and an OLE part to be presented separately. The design of the DB part, as shown in Supplementary Fig. 1, was composed of a screening/washout period (1–4 weeks), single-blind active run-in period (4 weeks), and a double-blind treatment period (8 weeks). Current antihypertensive medications that required tapering off were tapered according to the manufacturer’s labeling and stopped within 2 weeks after the initiation visit of the run-in period.

The key inclusion criteria for the study were Japanese male or female outpatients with essential hypertension, ≥18 years at the time of providing consent, untreated or currently taking antihypertensive therapy. Untreated patients (either newly diagnosed with essential hypertension or not having received antihypertensive drugs for 4 weeks prior to screening visit) required a mean sitting systolic blood pressure (msSBP) of ≥150 mmHg and <180 mmHg at both screening and run-in visit; treated patients (taking antihypertensive drugs within 4 weeks prior to screening visit) required msSBP <180 mmHg at screening visit, and msSBP ≥150 mmHg and <180 mmHg at run-in visit. Patients not adequately responsive to SacVal 200 mg treatment required msSBP ≥140 mmHg and <180 mmHg at the end of run-in/randomization visit (Day 1). Full inclusion criteria and major exclusion criteria are listed in Supplementary Table 1.

Eligible patients who successfully completed the run-in period and met BP criteria were randomized to one of the following four treatment arms in a ratio of 1:1:1:1 on Day 1 of the double-blind treatment period: SacVal 200 mg monotherapy; free combination of SacVal/AML 200 mg/2.5 mg; SacVal/AML 200 mg/5 mg; and SacVal/AML 200 mg/10 mg. Randomization was performed using a block size of 8 without stratification. The total study population consisted of approximately 180 patients per treatment arm, and an ABPM subset was conducted in approximately 120 patients per arm (Supplementary Fig. 2).

The study protocol was reviewed by the Independent Ethics Committee or Institutional Review Board for each study site. The study was conducted according to International Council for Harmonisation of Technical Requirements for Registration of Pharmaceuticals for Human Use E6 Guideline for Good Clinical Practice, which is based on the Declaration of Helsinki. Written informed consent was obtained from each patient at screening before any study-related procedure was performed.

### Study interventions

SacVal 200 mg was provided as tablets in an open-label box to all patients and AML 0 mg, 2.5 mg, 5 mg, and 10 mg as 4 capsules (placebo capsule or AML 2.5 mg capsule) in a blinded fashion (i.e., 4 placebo capsules for the SacVal 200 mg monotherapy arm and 4 AML 2.5 mg capsules for the SacVal/AML 200 mg/10 mg arm). Patients assigned in the SacVal/AML 200 mg/10 mg arm received 200 mg/5 mg for a week, and were then forced titrated to 200 mg/10 mg at Week 1 (Supplementary Fig. 1). All concomitant therapies were recorded on Case Report Forms and individually assessed against all exclusion criteria and any prohibited medications, particularly antihypertensives, which were tapered off and stopped within 2 weeks after starting the run-in period. No rescue antihypertensive medications were allowed but, if the patient discontinued the study drug permanently, conventional antihypertensive drugs were permitted until the end of the DB part.

### Study measurements

Baseline values for all efficacy and safety parameters were defined as the last non-missing assessment prior to the first dose of double-blind study medication. Efficacy was assessed in terms of office BP measurements and ambulatory blood pressure monitoring (ABPM) assessment. Office BP measurements at trough (24 ± 2 h post-dose) were made at each study visit in the morning (between 7:00 a.m. and 10:00 a.m.) while the patient was sitting by an automated digital BP device provided by the sponsor. msSBP and mean sitting diastolic blood pressure (msDBP) were calculated from the average of three separate readings taken 1–2 min apart.

ABPM over a 24-h period was conducted at randomization (but prior to the first dose of double-blind medication) and at the Week 8 visit in a subset of patients. BP and pulse rate were measured automatically using the ABPM device every 20 min during the day and every 30 min during the night. Daytime mean was defined as the average from self-reported time of waking to self-reported time of falling asleep; nighttime mean as the average from self-reported time of falling asleep to self-reported time of waking; morning mean as the average within 2 h after waking ( ≥ waking time to < waking time + 2 h).

Safety was assessed by adverse event (AE) monitoring, physical examinations, vital signs, electrocardiograms (ECGs), and laboratory analysis (hematology, blood chemistry, and urinalysis). AEs were graded as mild, moderate, and severe in terms of interference with normal activities.

### Study endpoints

The primary endpoint was the change from baseline at Week 8 in msSBP (office BP). Key secondary endpoints were the change from baseline at Week 8 in 24-h maSBP and the proportion of patients achieving BP control (office BP, <140/90 mmHg) at Week 8. Other secondary endpoints were the change from baseline at Week 8 in msDBP, maDBP and daytime/nighttime and morning maSBP. Other main exploratory endpoints of interest were the proportion of patients achieving BP target (office BP, <130/80 mmHg) and BP control during daytime, nighttime and morning at Week 8.

### Statistical analyses

The planned sample size was 154 completed patients per arm for SacVal/AML 200 mg/2.5 mg vs SacVal 200 mg monotherapy, assuming a true treatment difference in msSBP of 5.5 mmHg (common SD 14.0 mmHg), providing 93% power at a two-sided 5% significance level (two-sample *t* test). Assuming larger treatment differences of 6.5 and 8.0 mmHg, powers were 98.0% and 99.9% for SacVal/AML 200 mg/5 mg and SacVal/AML 200 mg/10 mg vs SacVal 200 mg monotherapy, respectively. The overall power for rejecting all three null hypotheses was 91%. Allowing for a 10% dropout rate, 688 participants (172 per arm) were planned for randomization. This sample size was also considered to provide adequate power for the analysis of key secondary endpoints. Details are provided in the Supplementary Methods.

Efficacy analysis were performed in the Full Analysis Set (FAS) per the intent-to-treat principle. The primary endpoint (change in msSBP) was analyzed using an analysis of covariance (ANCOVA) model with treatment as a factor and baseline msSBP as a covariate; change in msDBP was analyzed similarly with baseline in msDBP. Change in 24-h maSBP/maDBP were analyzed using repeated-measures ANCOVA model with treatment and post-dosing hours as factors, baseline 24-h maSBP/maDBP as a covariate and treatment-by-post-dosing hour interaction. BP control/target were analyzed using logistic regression model with treatment as factor and baseline msSBP as a covariate. Change in daytime/nighttime maSBP were analyzed using a repeated-measure ANCOVA model with treatment and time (daytime or nighttime) as factors, baseline 24-h maSBP as a covariate and treatment-by-time interaction. Change in morning maSBP was analyzed using ANCOVA model with treatment as factor and baseline morning maSBP as covariate. BP control during daytime/nighttime and morning were analyzed using a logistic regression model with treatment as factor and baseline 24-h maSBP (for daytime/nighttime) or baseline morning maSBP (for morning) as a covariate. Hypothesis testing was performed at an alpha level of 0.05 using a two-sided test. Least square (LS) mean and standard error, LS mean difference or conditional odds ratio compared to SacVal 200 mg arm and the corresponding 95% confidence intervals, and two-sided p-values for pairwise comparisons were also calculated. For endpoints obtained from ABPM, the ABPM subset was used for analysis.

Multiplicity adjustment for the primary and key secondary endpoints was performed using a hierarchical testing procedure to control the overall type I error rate at 0.05 [18].

Missing data were imputed based on clinical questions of interest for each endpoint. Details of the imputation methods are provided in Supplementary Methods.

A subgroup analysis of the primary endpoint was conducted using the ANCOVA model with treatment-by-subgroup interaction along with treatment as a factor and baseline msSBP as covariate. LS mean difference vs the SacVal 200 mg arm with 95% CIs were graphically presented by forest plot. The subgroups were defined by age ( < 65, ≥65 years); sex (male, female); baseline msDBP ( < 90, ≥90 mmHg); baseline BP status (low [grade 1]: 140 to <160 mmHg msSBP and/or 90 to <100 mmHg msDBP, high [grade 2]: 160 to <180 mmHg msSBP and/or 100 to <110 mmHg msDBP; patients meeting both were classified as high); diabetes (Yes, No); and estimated glomerular filtration rate (eGFR) ( < 30, 30 to <60, 60 to <90, ≥90 mL/min/1.73m^2^).

All statistical analyses were conducted using SAS version 9.4 (SAS Institute Inc., Cary, NC, USA) or higher and R version 4.1.0 (https://www.R-project.org/) or higher.

## Results

### Patients

In total, 1839 patients were screened and 1107 (60.2%) completed screening and entered the run-in period, of which 717 (64.8%) completed the run-in period and were randomized and received double-blind treatment representing the FAS for the analysis. A total of 390 (35.2%) patients discontinued the run-in period while 353 (31.9%) patients did not meet the randomization criteria, mainly due to the inclusion criteria defining inadequate response to SacVal (msSBP ≥140 mmHg) at the end of the run-in period (Supplementary Fig. 2).

Most patients (*n* = 707, 98.5%) completed the planned study phase and a high proportion (*n* = 684, 95.3%) completed study treatment while 33 patients (4.6%) discontinued study treatment before the end of the planned treatment phase, mainly due to AEs (*n* = 19, 2.6%).

All treatment arms were considered well balanced, including the ABPM subset, in terms of demographics, medical histories, and baseline disease characteristics (Table 1). Overall, the mean age was 61.1 years, approximately one-third of patients were >65 years, most patients (76.7%) were male, and the mean BMI was 25.67 kg/m^2^. The mean duration of hypertension was 9.6 years, and most patients (73.2%) had been treated with at least one antihypertensive medications within 4 weeks prior to screening (Supplementary Table 2). Renal function was moderately decreased at baseline (eGFR 30 to <60.0 mL/min/1.73m^2^) in approximately one-third of patients.

**Table 1:** Demographics, medical histories, and baseline disease characteristics

Characteristic	SacVal 200 mg	SacVal/AML 200 mg/2.5 mg	SacVal/AML 200 mg/5 mg	SacVal/AML 200 mg/10 mg	Total
	(N = 180)	(N = 180)	(N = 178)	(N = 179)	(N = 717)
Age, years, mean (SD)	62.7 (8.96)	60.5 (8.80)	60.5 (9.22)	60.6 (9.75)	61.1 (9.22)
Age category, n (%)					
<65 years	106 (58.9)	125 (69.4)	119 (66.9)	118 (65.9)	468 (65.3)
≥65 years	74 (41.1)	55 (30.6)	59 (33.1)	61 (34.1)	249 (34.7)
Sex, male, n (%)	145 (80.6)	143 (79.4)	138 (77.5)	124 (69.3)	550 (76.7)
Body mass index, kg/m^2^, mean (SD)	25.36 (3.722)	25.61 (3.818)	25.83 (3.910)	25.87 (4.394)	25.67 (3.966)
eGFR group, n (%)					
<30 mL/min/1.73 m^2^	0	0	0	0	0
≥30 mL/min/1.73 m^2^ to <60 mL/min/1.73 m^2^	66 (36.7)	63 (35.0)	57 (32.0)	62 (34.6)	248 (34.6)
≥60 mL/min/1.73 m^2^ to <90 mL/min/1.73 m^2^	108 (60.0)	108 (60.0)	115 (64.6)	111 (62.0)	442 (61.6)
≥90 mL/min/1.73 m^2^	6 (3.3)	9 (5.0)	6 (3.4)	6 (3.4)	27 (3.8)
History of dyslipidemia, n (%)	97 (53.9)	99 (55.0)	89 (50.0)	83 (46.4)	368 (51.3)
History of hyperuricemia, n (%)	42 (23.3)	49 (27.2)	50 (28.1)	42 (23.5)	183 (25.5)
History of diabetes, n (%)	30 (16.7)	26 (14.4)	25 (14.0)	14 (7.8)	95 (13.2)
Smoking status, n (%)					
Never	68 (37.8)	83 (46.1)	77 (43.3)	79 (44.1)	307 (42.8)
Current	32 (17.8)	29 (16.1)	37 (20.8)	30 (16.8)	128 (17.9)
Former	80 (44.4)	68 (37.8)	64 (36.0)	70 (39.1)	282 (39.3)
Duration of hypertension, years, mean (SD)	10.8 (9.68)	8.7 (7.24)	9.7 (7.27)	9.1 (8.28)	9.6 (8.19)
Antihypertensive drug treatment status, n (%)					
Treated	137 (76.1)	122 (67.8)	134 (75.3)	132 (73.7)	525 (73.2)
Untreated	43 (23.9)	58 (32.2)	44 (24.7)	47 (26.3)	192 (26.8)
Number of antihypertensive drug class (ATC class) used as prior medication taken within 4 weeks prior to screening visit, n (%)					
0	43 (23.9)	58 (32.2)	44 (24.7)	47 (26.3)	192 (26.8)
1	65 (36.1)	49 (27.2)	58 (32.6)	59 (33.0)	231 (32.2)
2	63 (35.0)	64 (35.6)	64 (36.0)	65 (36.3)	256 (35.7)
>2	9 (5.0)	9 (5.0)	12 (6.7)	8 (4.5)	38 (5.3)
msSBP, mmHg, mean (SD)	150.67 (8.610)	147.69 (7.303)	150.04 (8.388)	148.54 (7.083)	149.23 (7.945)
msDBP, mmHg, mean (SD)	94.39 (8.127)	96.26 (7.738)	96.54 (7.763)	95.89 (8.812)	95.77 (8.147)
Sitting pulse, beats/min, mean (SD)	73.2 (11.54)	75.7 (10.76)	74.0 (10.18)	74.2 (11.91)	74.3 (11.13)
24-h maSBP, mean (SD)	143.91 (13.635)	143.39 (13.677)	145.13 (13.438)	143.71 (13.419)	144.03 (13.518)
24-h maDBP, mean (SD)	86.32 (9.034)	87.07 (10.696)	88.64 (10.005)	86.89 (9.767)	87.23 (9.910)
Daytime maSBP, mean (SD)	148.14 (14.190)	147.94 (13.459)	150.05 (14.362)	148.54 (13.638)	148.66 (13.894)
Nighttime maSBP, mean (SD)	129.78 (15.524)	129.30 (16.855)	129.55 (15.426)	129.15 (15.089)	129.44 (15.701)
Morning maSBP, mean (SD)	148.04 (14.732)	149.90 (16.332)	148.91 (17.011)	148.56 (14.870)	148.86 (15.738)
Dipping status, n (%)					
Dipper	71 (39.4)	80 (44.4)	82 (46.1)	83 (46.4)	316 (44.1)
Non-dipper	50 (27.8)	47 (26.1)	41 (23.0)	41 (22.9)	179 (25.0)

A participant’s daytime mean maSBP was the average from self-reported time of waking to self-reported time of falling asleep; the nighttime mean maSBP was the average from the time of falling asleep to the time of waking; and the morning mean maSBP was the average within 2 h from the time of waking

*eGFR* estimated glomerular filtration rate, *maDBP* mean ambulatory diastolic blood pressure, *maSBP* mean ambulatory systolic blood pressure, *msDBP* mean sitting diastolic blood pressure, *msSBP* mean sitting systolic blood pressure, *SD* standard deviation

### BP-lowering effects

**Fig. 1:**
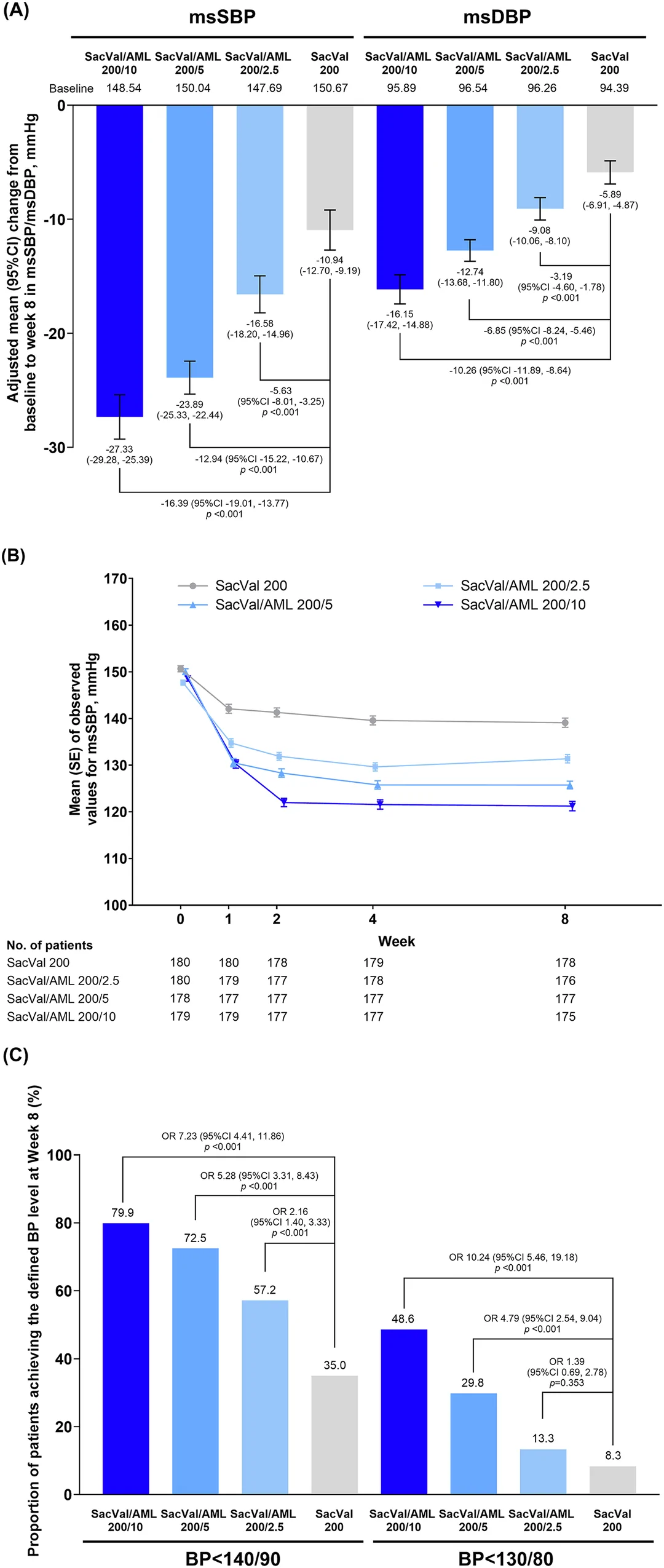
Efficacy results for office trough BP in the morning in terms of (**A**) change from baseline at Week 8 in msSBP and msDBP, (**B**) msSBP of observed values by visit up to Week 8, and (**C**) proportion of patients achieving the defined BP level at Week 8

In terms of the primary efficacy endpoint, all combination arms of SacVal/AML 200 mg/10 mg, 200 mg/5 mg, and 200 mg/2.5 mg demonstrated statistically significant superiority over the SacVal 200 mg monotherapy arm. The treatment difference (95%CI) in adjusted mean change from baseline in msSBP was −16.39 ( −19.01, −13.77), −12.94 ( −15.22, −10.67), and −5.63 ( −8.01, −3.25) mmHg favoring the SacVal/AML 200 mg/10 mg, 200 mg/5 mg, and 200 mg/2.5 mg arms, respectively (all p < 0.001 vs SacVal monotherapy arm) (Fig. 1(A)). All observed values for msSBP were consistently lower from Week 1 in the SacVal/AML combination arms and the clear dose-dependent effect was apparent by Week 2 and sustained through to Week 8 (Fig. 1(B)). Patients in the SacVal/AML 200 mg/10 mg arm received 200 mg/5 mg for a week, and were then forced titrated to 200 mg/10 mg at Week 1.

As a key secondary endpoint, the proportion of patients achieving BP control of <140/90 mmHg after 8 weeks of treatment was also statistically significant in favor of SacVal/AML combination arms in a dose-dependent manner (Fig. 1(C)). The proportion of patients achieving target BP of <130/80 mmHg after 8 weeks of treatment was greater in the SacVal/AML 200 mg/10 mg and 200 mg/5 mg combination arms compared with the SacVal 200 mg monotherapy arm (Fig. 1(C)).

**Fig. 2:**
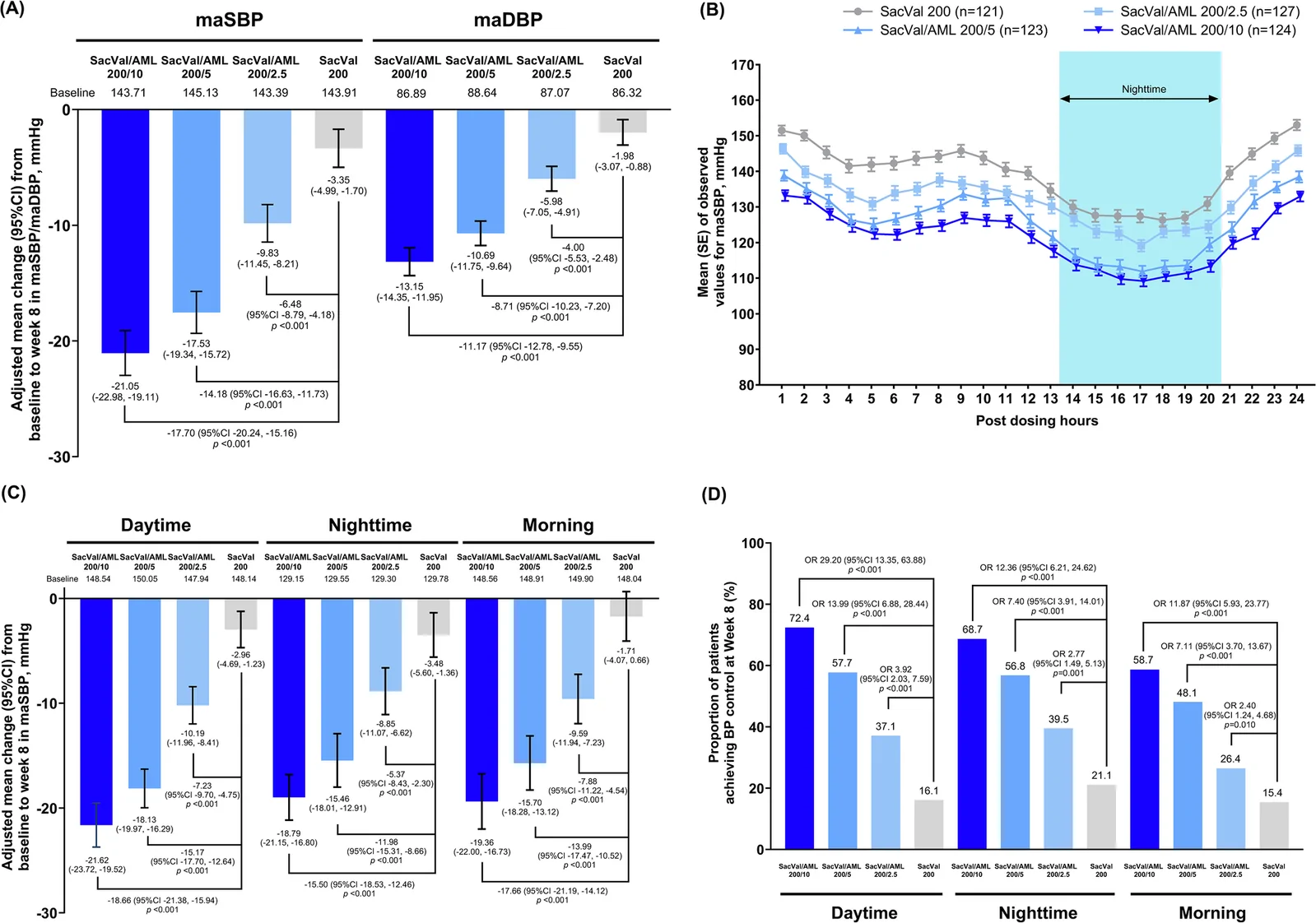
Efficacy results for ambulatory BP in terms of (**A**) change from baseline at Week 8 in maSBP and maDBP, (**B**) post-dosing hourly maSBP, (**C**) change from baseline at Week 8 in daytime, nighttime, and morning maSBP at Week 8, and (**D**) proportion of patients achieving BP control during daytime (SBP/DBP < 135/85 mmHg), nighttime (SBP/DBP < 120/70 mmHg) and morning (SBP/DBP < 135/85 mmHg). Note: For parts (**C**) and (**D**), a participant’s daytime mean was the average from self-reported time of waking to self-reported time of falling asleep; the nighttime mean was the average from the time of falling asleep to the time of waking; and the morning mean was the average within 2 h from the time of waking

In the other key secondary endpoint with the ABPM subset, a dose-dependent BP-lowering effect was observed and the change was greater in SacVal/AML combination arms compared with the SacVal 200 mg monotherapy arm for maSBP and maDBP (Fig. 2(A)). The adjusted mean (95%CI) for difference between treatment arms (vs SacVal 200 mg) in change from baseline at Week 8 in 24-h maSBP (mmHg) was −6.48 ( −8.79, −4.18), –14.18 ( −16.63, −11.73) and –17.70 ( −20.24, −15.16) for SacVal/AML 200 mg/2.5 mg, 200 mg/5 mg and 200 mg/10 mg respectively, which were all statistically significant (p < 0.001). BP-lowering effects by SacVal/AML were observed over 24 h (Fig. 2(B)). The differences observed between SacVal/AML combination arms and SacVal 200 mg monotherapy arm were present across the morning, daytime and nighttime periods (Fig. 2(C), (D)).

**Fig. 3:**
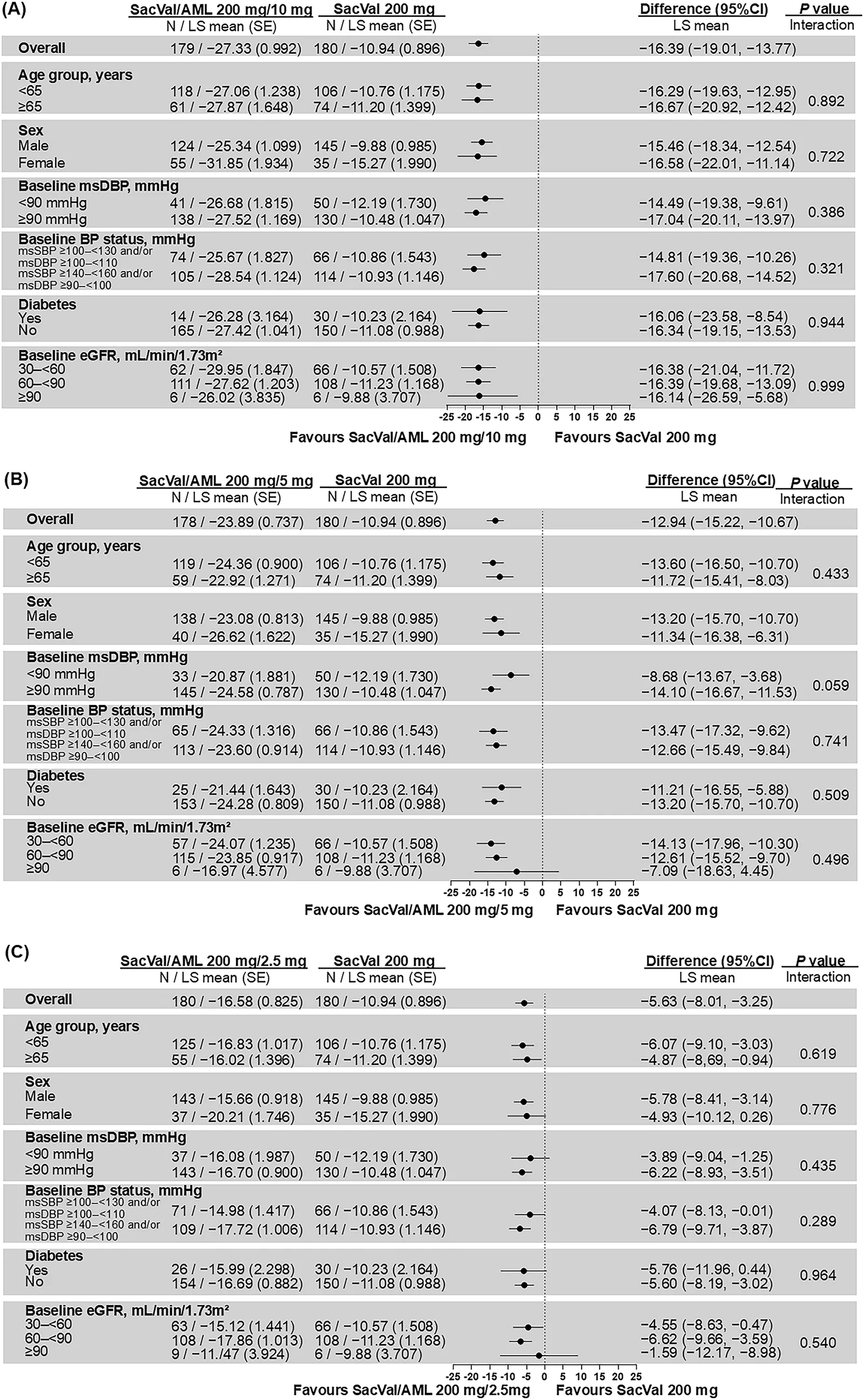
Forest plot of change from baseline at Week 8 in msSBP by subgroup in (**A**) SacVal/AML 200 mg/10 mg, (**B**) SacVal/AML 200 mg/5 mg, and (**C**) SacVal/AML 200 mg/2.5 mg arms

In the subgroup analysis, it was clear that greater BP-lowering effects for SacVal/AML arms vs SacVal 200 mg monotherapy arms were consistent across subgroups related to age, sex, baseline msDBP, baseline BP status, presence of comorbid diabetes, and baseline eGFR level (Fig. 3).

**Fig. 4:**
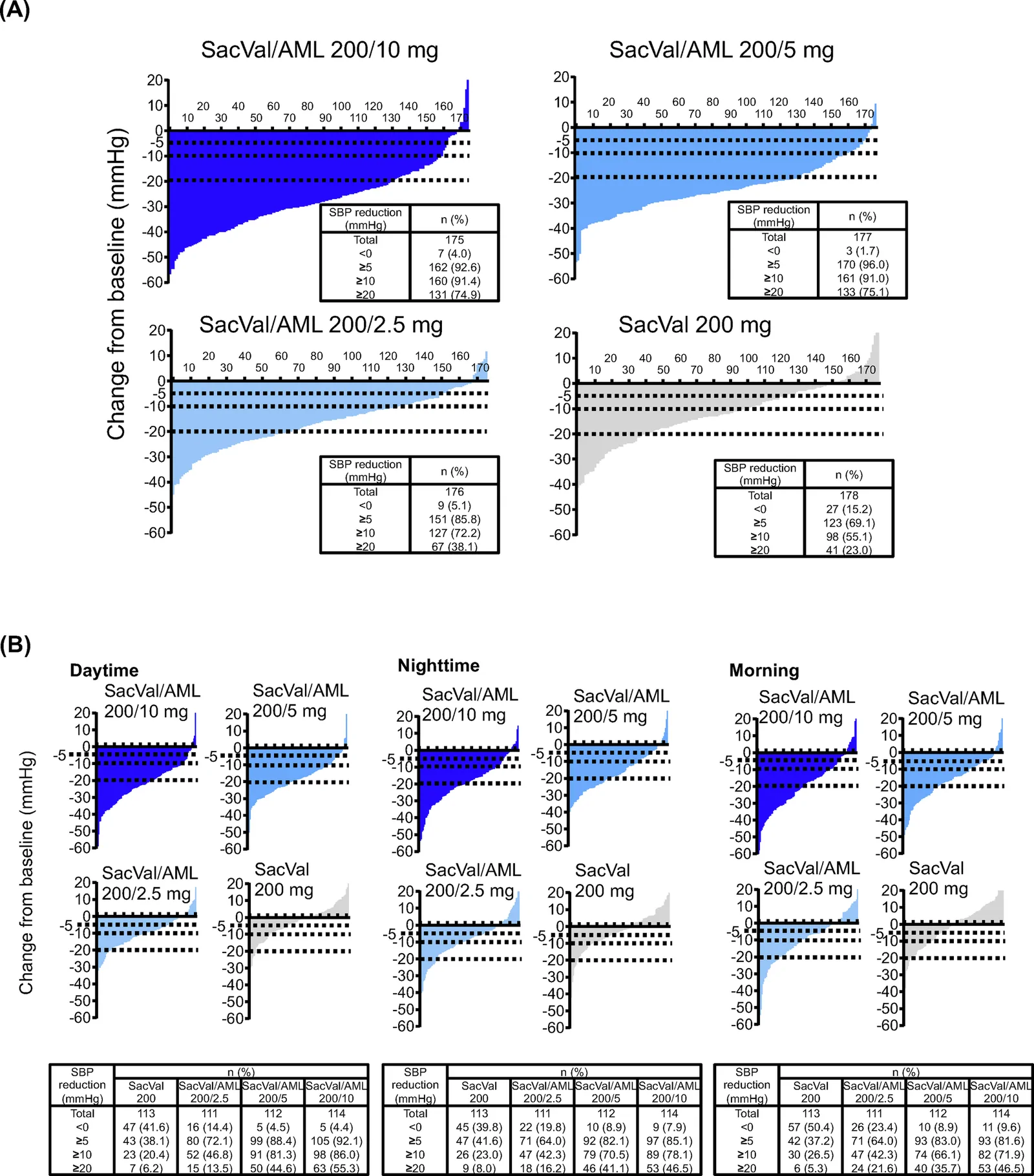
Waterfall plot change from baseline at Week 8 in (**A**) trough msSBP in the morning (**B**) maSBP during daytime, nighttime and morning. Note: A participant’s daytime mean was the average from self-reported time of waking to self-reported time of falling asleep; the nighttime mean was the average from the time of falling asleep to the time of waking; and the morning mean was the average within 2 h from the time of waking

Finally, broad efficacy in nearly all individual patients was shown based on the results of Waterfall plots both in office trough SBP in the morning and ambulatory SBP at each time period (daytime, nighttime, morning) (Fig. 4); the waterfall plots were analysed and the proportion of patients with BP reduction were assessed post hoc.

### Safety

The incidences of treatment-emergent AEs overall were similar across the treatment groups (range 25.3–31.3%), and most were deemed neither treatment-related nor serious (Table 2). Most AEs reported during the double-blind treatment period were of mild severity. However, treatment-related AEs were more frequent in the SacVal/AML 200 mg/10 mg arm (7.8%) compared with the other treatment arms (0.6–3.9%) as well as a higher frequency of AEs leading to treatment discontinuation (4.5% vs 0.6–2.2%)*.* The most common AE was nasopharyngitis (5.6–8.9%) while hypotension showed the greatest difference in incidence between the SacVal/AML 200 mg/10 mg arm (2.2%) and the other arms (0–0.6%). This was also reflected by generally greater proportions of patients in the SacVal/AML 200 mg/10 mg arm having hypotension-related events, such as postural dizziness (Supplementary Table 3). The incidence of peripheral edema was 0.6% in the SacVal/AML 200 mg/10 mg arm (Table 2).

**Table 2:** Overview of treatment emergent adverse events, most frequent (≥2% in any treatment group) treatment-emergent adverse events by preferred term, and safety topics (hypotension related and peripheral edema)

Category	SacVal 200 mg	SacVal/AML 200 mg/2.5 mg	SacVal/AML 200 mg/5 mg	SacVal/AML 200 mg/10 mg
	(N = 180) n (%)	(N = 180) n (%)	(N = 178) n (%)	(N = 179) n (%)
Overview of treatment-emergent AEs				
Adverse events	49 (27.2)	53 (29.4)	45 (25.3)	56 (31.3)
Treatment-related	1 (0.6)	7 (3.9)	2 (1.1)	14 (7.8)
SAEs	4 (2.2)	2 (1.1)	0	0
AEs leading to treatment discontinuation	3 (1.7)	4 (2.2)	1 (0.6)	8 (4.5)
Most frequent (≥2% in any treatment group) AEs				
Nasopharyngitis	16 (8.9)	11 (6.1)	10 (5.6)	14 (7.8)
Eczema	0	3 (1.7)	6 (3.4)	4 (2.2)
Hypotension	1 (0.6)	0	0	4 (2.2)
Dermatitis contact	1 (0.6)	4 (2.2)	2 (1.1)	1 (0.6)
Safety topic				
Hypotension-related AEs	2 (1.1)	4 (2.2)	2 (1.1)	8 (4.5)
Peripheral edema	0	1 (0.6)	0	1 (0.6)

Preferred terms are sorted by descending frequency in the SacVal/AML 200 mg/10 mg column. Preferred terms for the safety topics “Hypotension-related AEs” and “Peripheral Edema” are listed in Supplementary Table 3

MedDRA Version 28.0

*AE* adverse event, *SAE* serious adverse event

Changes in pulse rate from baseline at Week 8 were minimal during both the daytime and nighttime (Supplementary Table 4; post hoc). Importantly, there was no apparent increase in pulse rate with the addition of AML with changes ranging from 0.39 to −1.13 bpm in the daytime and from 0.33 to −0.69 bpm in the nighttime for AML-containing combinations arms (vs 0.60 and 0.66 for daytime and nighttime, respectively, in the SacVal monotherapy arm). There were no clinically significant changes in any of the laboratory parameters related to renal function, potassium, and glucose and lipid metabolism (Supplementary Table 5). A tendency towards reduction from baseline in serum uric acid was observed in all arms.

## Discussion

This multicenter, randomized, double-blind, parallel-group, active-controlled study is the first to evaluate the efficacy of a combination of SacVal 200 mg and AML 2.5/5/10 mg, compared to SacVal 200 mg monotherapy in patients with grade I or II hypertension inadequately controlled with SacVal 200 mg monotherapy. The study met its primary and key secondary objectives for all SacVal/AML combination arms. In addition, the study demonstrated dose dependency of SacVal/AML and BP-lowering effects were observed as early as Week 1–2 and sustained until Week 8.

A consistent and broadly applicable therapeutic effect across the study population was shown. SBP reduction by 5 mmHg and 10 mmHg is reported to be associated with a reduction in the risk of major cardiovascular events by about 10% and 20%, respectively [19, 20]. Treatment differences for the primary endpoint, change from baseline at week 8 in msSBP, were statistically significant and clinically meaningful for all SacVal/AML combinations vs SacVal monotherapy (all p < 0.001), with each treatment difference exceeding 5 mmHg. Treatment effects were also consistent across endpoints. These favorable efficacy results in the SacVal/AML combination arms were consistent across all evaluated patient subgroups, including by age, sex, and comorbidities. In this study, around 35% of patients had moderately decreased renal function (eGFR 30 to <60.0 mL/min/1.73m^2^) at baseline. Further, broad efficacy in nearly all individual patients, 91.4%, 91.0% and 72.2% of patients treated with SacVal/AML 200 mg/10 mg, 200 mg/5 mg, and 200 mg/2.5 mg, respectively, showed at least a 10 mmHg SBP reduction (Fig. 4(A), msSBP Waterfall plot).

SacVal/AML also showed high BP control and target BP achievement rates in patients not adequately controlled by 4 weeks of SacVal monotherapy. The proportions of patients achieving BP control ( < 140/90 mmHg) at Week 8 in the SacVal/AML 200 mg/10 mg, 200 mg/5 mg and 200 mg/2.5 mg arms were high at 79.9%, 72.5% and 57.2%, respectively (vs 35.0% in the SacVal monotherapy arm). Of note, the proportion of patients achieving BP target ( < 130/80 mmHg) at Week 8 was greater in the SacVal/AML 200 mg/10 mg and 200 mg/5 mg arms compared with the SacVal 200 mg arm (48.6% and 29.8%, respectively, vs. 8.3%). According to a previous report by Asayama et al. in 2019, the proportion of Japanese patients who achieve this target BP level was only approximately 20% [3]. In hypertensive patients initiating antihypertensive therapy, when the achievement rate of the target BP by the core metric [21] was evaluated, the achievement rate was only 26.7% [22]. Therefore, the achievement rate of target BP ( < 130/80 mmHg) with SacVal/AML 200 mg/10 mg and 200 mg/5 mg in patients not adequately controlled by SacVal monotherapy can be considered high and clinically meaningful.

Results of the ambulatory BP assessment demonstrated sustained 24-h antihypertensive effect of SacVal/AML combination treatment on maSBP and maDBP. Notably, BP reduction and improvement in BP control rate were observed during nighttime, morning, and daytime, although BP control during nighttime and morning is generally insufficient compared to daytime even with the use of multiple antihypertensive medications [6]. The importance of managing both morning and nocturnal BP has been emphasized in Asian populations, as the increased morning and nocturnal BP are associated with cardiovascular events and a tendency towards these issues may be higher in Asians than in Western population [23–25]. SacVal/AML combination treatment may contribute to reduce the risk of cardiovascular events through sustained 24-h BP-lowering effect in Japanese patients.

Overall, these findings suggest that, in addition to the widely used combination of ARB and CCB in Japan, the combination of ARNI and CCB may be considered a potential treatment option for uncontrolled hypertension in Japanese patients.

There were no deaths during the double-blind treatment period. All AEs observed in this study were consistent with those expected with SacVal or AML treatment, and no new safety signals were identified. Overall, treatment-emergent AEs in the double-blind period were mostly mild in severity and the incidences of total AEs were similar among all groups. Treatment-related AEs and AEs leading to treatment discontinuations were also similar across SacVal monotherapy, SacVal/AML 200 mg/2.5 mg, and 200 mg/5 mg arms, and slightly higher with SacVal/AML 200 mg/10 mg arm, mainly due to hypotension and dizziness. In the SacVal/AML 200 mg/10 mg arm, patients received SacVal/AML 200 mg/5 mg for 1 week and were then force titrated to 200 mg/10 mg at Week 1, irrespective of BP response at the lower dose. Importantly, the higher incidence of hypotension-related AEs and treatment discontinuations in the 200 mg/10 mg arm is likely related to this mandatory up-titration strategy, and the associated BP-lowering effects. These findings were consistent with the known safety profile of the study treatment. However, caution with the highest dose combination of SacVal/AML 200 mg/10 mg may be prudent in certain groups prone to low BP effects (e.g., the elderly).

Peripheral edema is known as a characteristic adverse reaction of AML at a dose of 10 mg and was observed in AML monotherapy study with Japanese hypertensive patients, in which the incidence of peripheral edema was higher in the AML 10 mg arm (5/151, 3.3%) compared to AML 5 mg arm (0/154, 0%) [26]. In our study, the incidence of peripheral edema was 0.6% in the SacVal/AML 200 mg/10 mg arm (Table 2), which was numerically lower than previously reported rates. Although differences in study design and patient populations limit direct comparison, the lower incidence observed in this study is consistent with a potential attenuation of peripheral edema with the SacVal/AML combination.

AML has been reported to increase pulse rate [27]. However, in this study, the addition of AML to SacVal did not result in an increase in pulse rate compared to SacVal alone. Despite having a diuretic effect, SacVal reduced serum uric acid as reported in a previous clinical study [28]. In our study, a similar trend was observed and the combination with AML tended to further lower serum uric acid compared to SacVal monotherapy (see Supplementary Table 5).

### Limitations

Since the study population was limited to patients with grade I or II hypertension who were inadequately controlled with SacVal 200 mg monotherapy, caution is warranted when generalizing the findings to patients with more severe hypertension. The 8-week study period was relatively short, limiting assessment of long-term efficacy and safety. A 52-week OLE part of this study should provide additional efficacy and safety information to address this. As the study was conducted in Japanese patients, the external validity (generalizability) of the findings to other ethnicities or geographic regions may be limited.

## Conclusions

This randomized, double-blind study demonstrated that combination treatment with SacVal/AML 200/10 mg, 200 mg/5 mg or 200 mg/2.5 mg was efficacious in a dose-dependent manner as well as safe and well-tolerated in patients with grade I and II hypertension not adequately controlled by SacVal 200 mg monotherapy. Based on the results from the DB part, these findings support SacVal/AML as a potent combination option for Japanese patients who do not reach target BP with SacVal monotherapy. Additional data from the OLE part are expected to further inform and support the efficacy and safety profile observed in the DB part.

## Supplementary information

Supplementary Materials

## Data Availability

Novartis is committed to sharing with qualified external researchers, access to patient-level data and supporting clinical documents from eligible studies. These requests are reviewed and approved by an independent review panel on the basis of scientific merit. All data provided is anonymized to respect the privacy of patients who have participated in the trial in line with applicable laws and regulations. This trial’s data availability accords with the criteria and process described at www.clinicalstudydatarequest.com.
