# Publisher Correction: Randomized-controlled trial of sacubitril/valsartan and amlodipine combination in Japanese uncontrolled hypertensive patients (J-SAMIT)

**DOI:** 10.1038/s41440-026-02739-w

**Published:** 2026-07-13

**Authors:** Kazuomi Kario, Hiromi Rakugi, Chiyo Ito, Sarfaraz Sayyed, Toshihito Kitamura, Yukina Tanaka, Ankur Malhotra

**Affiliations:** 1 Division of Cardiovascular Medicine, Department of Medicine, Jichi Medical University School of Medicine, Tochigi, Japan; 2 Osaka Rosai Hospital, Sakai, Osaka Japan; 3 Novartis Pharma K.K., Tokyo, Japan; 4 Novartis Healthcare Private Limited, Mumbai, India

Correction to: *Hypertension Research*

https://doi.org/10.1038/s41440-026-02704-7, published online 30 June 2026

Fig.3 in the original xml version of this article was incorrectly enlarged and cut-off. The original article has been corrected.

