# Supplementary material for: Randomized-controlled trial of sacubitril/valsartan and amlodipine combination in Japanese uncontrolled hypertensive patients (J-SAMIT)
Source: Hypertens Res. 2026 Jun 30;49(9):2587–600. doi: 10.1038/s41440-026-02704-7 (PMC13547103; doi:10.1038/s41440-026-02704-7)
Supplement: Supplementary file 1 [file 41440_2026_2704_MOESM1_ESM.docx]

# **Supplementary Materials**

Supplementary Methods………………………………………………………………………………………………………2

Supplementary Table 1. Inclusion and exclusion criteria………………………………………………………..4

Supplementary Table 2. Prior antihypertensive medication taken within 4 weeks prior to screening visit…………………………………………………………………………………………………………………….…7

Supplementary Table 3. Preferred terms comprising the safety topics ‘Hypotension-related AEs’ and ‘Peripheral edema’……………………………………………………………………………………………….…8

Supplementary Table 4. Pulse rate change from baseline at Week 8…………………………………….9

Supplementary Table 5. Clinical laboratory evaluation…………………………………………………………11

Supplementary Figure 1. Study design…………………………………………………………………………………13

Supplementary Figure 2. Patient disposition…………………………………………………………………….…14

# **Supplementary Methods**

# **Sample size for key secondary endpoints**

The planned sample size was considered sufficient for the change from baseline at Week 8 in 24-hour maSBP and the proportion of patients achieving BP control at Week 8. For 24-hour maSBP, 384 completed participants (96 per arm) were targeted, assuming a true treatment difference of 4.5 mmHg and SD of 9.0 mmHg, providing 93% power at a two-sided significance level (two-sample t-test). Allowing for 20% attrition/invalid ABPM, this corresponded to 480 randomized participants (120 per arm). For BP control, 172 participants per arm provided 90% power (two-sided 5% level), assuming BP control rate of 38% for combination therapy and 22% for monotherapy and treating dropouts as non-responders.

# **Missing data**

For msSBP/msDBP, 24-hour maSBP/maDBP and daytime/nighttime/morning maSBP, missing data in the SacVal/AML combination arms after observing the intercurrent events (study treatment discontinuation and/or use of other antihypertensive medication) were multiply imputed using observed data from the SacVal monotherapy arm, under the assumption that patients with missing data have outcomes similar to those in the SacVal monotherapy arm (jump-to-reference imputation technique). Missing data in the SacVal monotherapy arm after intercurrent events were multiply imputed using observed data from the same arm, under the missing-at-random (MAR) assumption. Missing data not related to intercurrent event were also multiply imputed using observed data from the same arm under a MAR assumption.

For BP control and BP target, missing data were singly imputed as non-responders. For BP control during daytime, nighttime and morning, missing data after intercurrent event were singly imputed as non-responders, whereas missing BP values not related to intercurrent events were multiply imputed under a MAR assumption and BP control was determined based on the imputed values.

## **Supplementary Table 1. Inclusion and exclusion criteria**

# **Inclusion criteria**

- Signed informed consent must be obtained prior to participation in the study
- Japanese male or female outpatients 18 years of age or older (at the time of signing informed consent)
- Patients with grade 1 and 2 essential hypertension, untreated or currently taking antihypertensive therapy
- Untreated patients (either newly diagnosed with essential hypertension or those with a history of hypertension but have not been taking any antihypertensive drugs for 4 weeks prior to screening visit) must have a msSBP of ≥150 mmHg and <180 mmHg at both screening and run-in visits
- Treated patients (taking antihypertensive drugs within 4 weeks prior to screening visit must have msSBP <180 mmHg at screening visit, and msSBP ≥150 mmHg and <180 mmHg at run-in visit
- Patients who are not adequately responsive to SacVal 200 mg treatment must have a msSBP ≥140 mmHg and <180 mmHg at the end of run-in/randomization visit
- Patients who are able to communicate well with the Investigator, to understand and comply with all study requirements, and demonstrate good medication compliance (≥ 80% compliance rate) during the single-blind active run-in period

# **Major Exclusion criteria**

- Patients currently on one or more antihypertensive medications in whom the Investigator considers that the medications cannot be safely discontinued for the duration of the DB part
- Administration of any agent indicated for the treatment of hypertension after the screening visit, with the permitted exception of those antihypertensive medications requiring tapering down commencing at screening visit and stopped within 2 weeks after Visit Run-in
- Severe hypertension (msSBP ≥180 mmHg and/or msDBP ≥110 mmHg at any visit prior to or at randomization), or malignant hypertension
- History or evidence of a secondary form of hypertension
- Patients with Type 1 or Type 2 diabetes mellitus not well controlled based on the Investigator’s clinical judgement
- Concomitant refractory angina pectoris
- Clinically significant valvular heart disease at screening
- History of transient ischemic attack (TIA), myocardial infarction, coronary bypass surgery, or any percutaneous coronary intervention (PCI) within 12 months prior to screening visit
- Previous or current diagnosis of heart failure (NYHA Class II-IV)
- History of angioedema, drug-related or otherwise, as reported by the patient
- Known active liver disease or cirrhosis or evidence of hepatic disease as determined by ALT or AST >2 times the upper limit of the normal (x ULN), or TBL >2 x ULN or Alkaline Phosphatase (ALP) >1.5 x ULN range at screening, or history of hepatic encephalopathy, history of esophageal varices, or history of portocaval shunt
- Evidence of renal impairment including but not limited to any one of the following: eGFR <30 mL/min/1.73 m^2^ at screening calculated by Japanese eGFR formula, history of dialysis, history of nephrotic syndrome, or history of renal transplantation
- Patients with night-shift employment (for patients participating in the ABPM subset)
- Pregnant or nursing (lactating) women
- Women of child-bearing potential, defined as all women physiologically capable of becoming pregnant, unless they are using highly effective methods of contraception while taking study treatment and for 10 days after stopping study treatment

## **Supplementary Table 2. Prior antihypertensive medication taken within 4 weeks prior to screening visit**

|  | **SacVal 200 mg  (N=180)** | **SacVal/AML  200 mg/2.5 mg (N=180)** | **SacVal/AML 200mg/5 mg (N=178)** | **SacVal/AML  200 mg/10 mg (N=179)** | **Total (N=717)** |
| --- | --- | --- | --- | --- | --- |
| Number of subjects with at least one medication | 137 (76.1) | 122 (67.8) | 134 (75.3) | 132 (73.7) | 525 (73.2) |
| Angiotensin II receptor blocker | 83 (46.1) | 77 (42.8) | 87 (48.9) | 80 (44.7) | 327 (45.6) |
| Angiotensin converting enzyme inhibitor | 1 (0.6) | 0 | 0 | 1 (0.6) | 2 (0.3) |
| Angiotensin receptor neprilysin inhibitor | 10 (5.6) | 12 (6.7) | 15 (8.4) | 12 (6.7) | 49 (6.8) |
| Beta blocker | 5 (2.8) | 7 (3.9) | 4 (2.2) | 4 (2.2) | 20 (2.8) |
| Calcium channel blocker | 111 (61.7) | 101 (56.1) | 101 (56.7) | 107 (59.8) | 420 (58.6) |
| Amlodipine | 71 (39.4) | 60 (33.3) | 71 (39.9) | 69 (38.5) | 271 (37.8) |
| Amlodipine besilate | 21 (11.7) | 23 (12.8) | 16 (9.0) | 18 (10.1) | 78 (10.9) |
| Thiazide diuretics | 6 (3.3) | 3 (1.7) | 10 (5.6) | 7 (3.9) | 26 (3.6) |
| Others | 3 (1.7) | 4 (2.2) | 5 (2.8) | 2 (1.1) | 14 (2.0) |

## **Supplementary Table 3. Preferred terms comprising the safety topics ‘Hypotension-related AEs’ and ‘Peripheral edema’**

| **Safety topics**  **Preferred term** | **SacVal 200 mg  N=180 n (%)** | **SacVal/AML  200 mg/2.5 mg N=180 n (%)** | **SacVal/AML  200 mg/5 mg N=178  n (%)** | **SacVal/AML  200 mg/10 mg  N=179  n (%)** |
| --- | --- | --- | --- | --- |
| Hypotension-related AEs | 2 (1.1) | 4 (2.2) | 2 (1.1) | 8 (4.5) |
| Hypotension | 1 (0.6) | 0 | 0 | 4 (2.2) |
| Dizziness | 1 (0.6) | 3 (1.7) | 1 (0.6) | 1 (0.6) |
| Dizziness postural | 0 | 1 (0.6) | 1 (0.6) | 2 (1.1) |
| Blood pressure decreased | 0 | 0 | 0 | 1 (0.6) |
| Loss of consciousness | 1 (0.6) | 0 | 0 | 0 |
| Peripheral edema | 0 | 1 (0.6) | 0 | 1 (0.6) |
| Edema peripheral | 0 | 1 (0.6) | 0 | 1 (0.6) |

Preferred terms are sorted by descending frequency in the SacVal/AML 200 mg/10 mg column.

MedDRA Version 28.0.

**Supplementary Table 4. Pulse rate change from baseline at Week 8**

|  | | **SacVal 200 mg  (N=121)** | | | **SacVal/AML  200 mg/2.5 mg (N=127)** | | | **SacVal/AML  200 mg/5 mg (N=123)** | | | **SacVal/AML  200 mg/10 mg (N=124)** | | |
| --- | --- | --- | --- | --- | --- | --- | --- | --- | --- | --- | --- | --- | --- |
| **Outcome** | **Statistics** | **Base** | **Post** | **Change** | **Base** | **Post** | **Change** | **Base** | **Post** | **Change** | **Base** | **Post** | **Change** |
| Daytime | n | 113 | 113 | 113 | 111 | 111 | 111 | 112 | 112 | 112 | 114 | 114 | 114 |
|  | Mean | 75.38 | 75.98 | 0.60 | 79.32 | 78.19 | −1.13 | 78.43 | 77.88 | −0.55 | 76.45 | 76.83 | 0.39 |
|  | SD | 8.807 | 9.173 | 6.408 | 9.274 | 8.683 | 6.289 | 10.095 | 9.812 | 5.612 | 10.062 | 9.681 | 5.649 |
|  | Median | 75.50 | 76.12 | 0.16 | 79.45 | 77.35 | −0.78 | 79.08 | 78.24 | −0.95 | 75.93 | 76.74 | 0.22 |
|  | Min | 55.5 | 54.2 | −17.3 | 57.6 | 58.6 | −20.2 | 48.0 | 56.7 | −29.2 | 53.2 | 51.5 | −18.8 |
|  | Max | 95.8 | 98.7 | 17.5 | 106.4 | 105.8 | 14.8 | 98.8 | 103.0 | 14.9 | 100.6 | 99.0 | 18.2 |
| Nighttime | n | 113 | 113 | 113 | 111 | 111 | 111 | 112 | 112 | 112 | 114 | 114 | 114 |
|  | Mean | 64.27 | 64.94 | 0.66 | 67.64 | 66.94 | −0.69 | 67.27 | 66.83 | −0.44 | 64.92 | 65.25 | 0.33 |
|  | SD | 9.121 | 9.310 | 6.327 | 9.582 | 8.905 | 7.303 | 9.905 | 9.446 | 7.655 | 10.043 | 9.142 | 7.218 |
|  | Median | 63.20 | 63.23 | 0.03 | 67.15 | 65.61 | −0.23 | 66.88 | 66.82 | 0.81 | 65.03 | 64.26 | −0.54 |
|  | Min | 47.8 | 46.9 | −14.7 | 48.3 | 49.0 | −38.9 | 44.6 | 45.5 | −29.4 | 42.4 | 43.8 | −21.2 |
|  | Max | 100.1 | 95.8 | 18.7 | 104.5 | 97.5 | 23.4 | 89.6 | 90.0 | 21.1 | 96.4 | 89.3 | 29.8 |

Unit: beats/min, Base = Baseline, Change = Post Baseline − Baseline.
At each time point, only patients with a value at both Baseline and that time point are included.
This analysis is performed on a subset of subjects with ABPM values at baseline in the FAS.
A participant’s daytime mean maSBP was the average from self-reported time of waking to self-reported time of falling asleep; the nighttime mean maSBP was the average from the time of falling asleep to the time of waking; and the morning mean maSBP was the average within 2 hours from the time of waking.

## **Supplementary Table 5. Clinical laboratory evaluation**

|  | **SacVal 200 mg  N=180** | **SacVal/AML  200 mg/2.5 mg N=180** | **SacVal/AML  200 mg/5 mg N=178** | **SacVal/AML  200 mg/10 mg N=179** |
| --- | --- | --- | --- | --- |
| Creatinine (mmol/L), serum |  |  |  |  |
| Baseline | 79.8 (15.16) | 79.3 (15.23) | 79.5 (16.76) | 78.5 (15.84) |
| Change from baseline at Week 8 | 0.3 (6.82) | −1.5 (6.63) | −1.7 (6.64) | −2.0 (7.04) |
| eGFR (mmol/L), serum |  |  |  |  |
| Baseline | 64.9 (12.11) | 65.9 (12.97) | 65.5 (12.74) | 64.8 (12.78) |
| Change from baseline at Week 8 | −0.1 (6.22) | 1.5 (5.90) | 1.9 (6.46) | 2.2 (6.25) |
| Blood urea nitrogen (mmol/L), serum |  |  |  |  |
| Baseline | 4.833 (1.1291) | 4.761 (1.0401) | 4.799 (1.2720) | 4.994 (1.2817) |
| Change from baseline at Week 8 | 0.079 (1.01017) | 0.087 (1.0665) | 0.161 (1.0268) | 0.113 (1.0718) |
| Potassium (mmol/L), serum |  |  |  |  |
| Baseline | 4.33 (0.317) | 4.30 (0.371) | 4.32 (0.352) | 4.30 (0.350) |
| Change from baseline at Week 8 | 0.00 (0.338) | 0.00 (0.324) | −0.03 (0.318) | −0.04 (0.314) |
| Fasting glucose (mmol/L), plasma |  |  |  |  |
| Baseline | 6.17 (1.420) | 5.91 (1.094) | 5.93 (1.293) | 5.83 (0.993) |
| Change from baseline at Week 8 | −0.03 (0.879) | 0.04 (0.749) | 0.02 (0.773) | 0.06 (0.550) |
| Total cholesterol (mmol/L), serum |  |  |  |  |
| Baseline | 5.355 (0.8149) | 5.253 (0.9545) | 5.361 (0.9437) | 5.206 (0.977) |
| Change from baseline at Week 8 | −0.067 (0.4805) | −0.024 (0.5475) | −0.053 (0.5683) | −0.027 (0.5946) |
| Triglycerides (mmol/L), serum |  |  |  |  |
| Baseline | 1.424 (0.7091) | 1.427 (0.7978) | 1.620 (1.2795) | 1.487 (1.0706) |
| Change from baseline at Week 8 | 0.102 (0.8020) | 0.082 (0.9027) | −0.052 (1.1794) | −0.034 (0.8798) |
| Uric acid (mmol/L), serum |  |  |  |  |
| Baseline | 345.6 (82.88) | 355.0 (81.89) | 352.8 (69.99) | 351.0 (75.20) |
| Change from baseline at Week 8 | −7.2 (44.65) | −15.4 (41.39) | −19.7 (46.10) | −32.7 (39.47) |

All values are mean (SD)

# **Supplementary Figure 1. Study design**

Note: ^a^Free‑combination: SacVal 200 mg tablet plus four AML capsules (a combination of AML 2.5 mg and placebo capsules to achieve AML 0, 2.5, 5, or 10 mg).

^b^Participants who returned one week after initial screening visit without meeting the blood pressure requirements for the single-blind period could be rescheduled for additional blood pressure checks at the Investigator’s discretion for up to three additional weeks at weekly intervals.

^c^Participant returned to the study site one week after screening visit (Visit Scr) and did not meet the blood pressure requirements for the single-blind period, the participant could be rescheduled for additional blood pressure checks one week after the visit at the Investigator’s discretion.

# **Supplementary Figure 2. Patient disposition**

Abbreviation: ABPM, ambulatory blood pressure monitoring.
Note: ^a^Includes one patient who was randomized in error despite being a run-in failure; ^b^ABPM subset comprises participants in the FAS with consent for ABPM and valid ABPM values at baseline.
